# “They have more than enough to do than patch up people like me.” Experiences of seeking support for self‐harm in lockdown during the COVID‐19 pandemic

**DOI:** 10.1111/jpm.12834

**Published:** 2022-04-24

**Authors:** Cara Sass, Kate Farley, Cathy Brennan

**Affiliations:** ^1^ Leeds Institute of Health Sciences University of Leeds Leeds UK

**Keywords:** COVID‐19, help‐seeking, mental health, online, qualitative, self‐harm

## Abstract

**What is known on the subject?:**

- In the initial months of the pandemic, there was no significant increase in demand for mental health services
- During the pandemic, there was an increase in people reporting an increase in suicidal thoughts.
- Understanding of the experience of seeking help for self‐harm during lockdown is lacking, in terms of availability and accessibility of support services

**What the paper adds to existing knowledge?:**

- People who self‐harm found that their support structures were significantly impacted by lockdown
- Lockdown presented relational difficulties for people who self‐harm

**What are the implications for practice?:**

- Learning about the experience of receiving support from mental health liaison services during lockdown from a first‐hand perspective is essential to improving the provision of these services in the future.
- In times of national crisis, services should be prepared to support clients via alternative means including telephone and online.
- Public facing messages about service availability should be carefully expressed to minimize misunderstandings.

**Abstract:**

**Introduction**

National lockdown caused disruption to health services and third‐sector organizations offering support to people who self‐harm. Early reports suggested self‐harm‐related hospital attendances declined. Lack of knowledge related to the availability and accessibility of support provisions for people who self‐harm warrants exploration into how they experienced help‐seeking during lockdown.

**Aims**

This study aimed to explore the experiences of people who self‐harm and perspectives on help‐seeking.

**Method**

We conducted a qualitative study based on telephone and email interviews with 14 people who self‐harm in England.

**Results**

Participants identified challenges to coping with life in lockdown, emphasizing the role of self‐harm. Help‐seeking was impeded by feeling like a burden and potential for spreading the virus. People who self‐harm exercised self‐reliance in response to “stay home” messaging, but some may have struggled without formal support. Online support served an important role in continuity of care during lockdown but could widen inequalities from limited resources and access.

**Implications for practice**

Helping mental health liaison nurses to understand the experiences of people who self‐harm during lockdown is critical to providing continuing support to this population. Services should consider how and when they communicate changes to their provisions to the public, and the impact this will have on those in need of support.

## 1 INTRODUCTION

Since the first wave of COVID‐19 spread throughout the UK, attention has been paid to the potential consequences of lockdown, economic adversity and physical health difficulties on mental health, especially for frontline workers (Lai et al., 2020). Clinicians specializing in self‐harm warned in early 2020 about pandemic‐associated risks to people with existing mental health conditions that could lead to greater inequity of healthcare (Gunnell et al., 2020; Jefsen et al., 2020; Moesmann Madsen et al., 2020). Those managing mental health conditions prior to the pandemic may have faced an exacerbation of their symptoms (Kaufman et al., 2020; Rains et al., 2021), owing to increased risk of negative consequences associated with lockdown measures. This may be precipitated by factors including financial and economic insecurity; limited professional support and social isolation; lack of activities to support stability, routine and health‐promoting behaviours; increases in problematic use of alcohol and substances; and harmful effects of quarantine measures on mental wellbeing (Brooks et al., 2020; Gunnell et al., 2020; Moesmann Madsen et al., 2020; Niederkrotenthaler et al., 2020). This emphasizes the importance of ensuring that access to support for self‐harm is consistent during times of crisis.

The national COVID social study has gathered data on mental health and suggested an increase in thoughts of self‐harm and suicide in the general population (Pierce et al., 2020). Self‐harm, defined as intentional acts of injury or poisoning to the body (Hawton et al., 2007), serves a multitude of functions which can be associated with the following: communicating distress to others; responding to negative thoughts or emotions; managing or controlling one's mental state; and pursuing positive feelings (Edmondson et al., 2016). The extent to which self‐harm meets these functions will vary between individuals. In the absence of intervention, a strong correlation exists between self‐harm and later suicide (Cooper et al., 2005; Hawton & Harriss, 2008; Owens et al., 2002).

Evidence to suggest a change in actual rates of self‐harm during the pandemic is lacking (Kapur et al., 2021), and anecdotal reports suggest an absence of the expected early surge of demand on mental health crisis provisions internationally (Johnson et al., 2020; Rains et al., 2021), including for self‐harm related presentations to hospitals in the UK (Hawton, Lascelles, et al., 2021).

In spite of this initial lull of activity seen by emergency services, healthcare workers have expressed concern that rates of self‐harm will increase due to the ongoing impact on psychological wellbeing. Analyses of previous major outbreaks suggest that we may be yet to observe the long‐term consequences of the pandemic for those with enduring mental health conditions (Cheung et al., 2008; Wasserman, 1992; Zortea et al., 2020). This may be precipitated by factors including economic insecurity; limited professional support and social isolation; lack of activities to support stability, routine and health‐promoting behaviours; increases in problematic use of alcohol and substances; and harmful effects of quarantine measures on mental wellbeing (Brooks et al., 2020; Gunnell et al., 2020; Moesmann Madsen et al., 2020; Niederkrotenthaler et al., 2020). This emphasizes the importance of ensuring that access to support for self‐harm is consistent during times of crisis.

Self‐harm related interactions with hospital‐based services in the UK are predominantly through hospital emergency departments (EDs) (Geulayov et al., 2016). During the pandemic, these services have been under additional strain and experienced reorganization and policy changes, in attempts to minimize transmission risk (Iqbal & Chaudhuri, 2020). Our informal discussions with user‐led services suggested that people who self‐harm were unsure of the services continuing to operate during lockdown, and even if they could identify resources, were reluctant to ask for support. The sharp decline in ED presentations with self‐harm may be due less to a reduction in acts of self‐harm, and more to factors including fear of transmission, uncertainty over availability of help, or expectations of stigma or of being a burden on over‐stretched services (Kapur et al., 2021). Early reports suggest that people with mental health needs re‐focused their attention on resources they perceived to be easier to access during lockdown (Gillard et al., 2021) such as helplines and online forums, having less contact with professionals than similar populations in previous years (Iob et al., 2020).

Third‐sector services also faced dramatic changes to provision, with the eradication of face‐to‐face support and group activities, and increased demand for support. This necessitated a shift to online and remote forms of communication for many organizations despite lacking the infrastructure and resources to support these changes. The COVID‐19 pandemic is a developing situation, and knowledge in relation to the nature and scale of support needs for people who self‐harm over this period is lacking. An informed understanding of service user perceptions of the support available to them during national lockdown will enable greater provision and appropriate organization of support for people who self‐harm during future national or global crises. To support the development of accessible services, this study explored the experiences of people who self‐harm during the first lockdown in March 2020, focusing on mental health impact and experiences of seeking help.

## 2 METHOD

The aims of this study were to understand the following:

- The impact of the lockdown on the day‐to‐day life of people who self‐harm
- People's help‐seeking behaviours during the first COVID‐19 lockdown in March 2020
- Changes to participants' support structures during the first lockdown

### 2.1 Design

This was a qualitative analysis of transcripts of semi‐structured interviews conducted by two researchers (CS and KF) via telephone and email. Telephone interviews followed a semi‐structured topic guide (see appendix) to explore any changes in the participant's situation and their experiences of seeking support related to self‐harm during lockdown. These questions were developed in collaboration with individuals with lived experience of self‐harm and a network of third‐sector partners, described further below. Participants could choose their mode of participation based on personal preference, in response to anecdotal feedback from third‐sector partners that audio recording may have deterred some individuals from taking part. Those requesting email interviews were given a choice of receiving an abridged version of the telephone guide and associated prompts in full, or one question at a time over multiple email exchanges with the same researcher. Expectations relating to response times, follow‐up questions as required and reminder emails were agreed with the participant in advance. Audio interviews were transcribed and the full set of data were analysed using Thematic Analysis (Braun & Clarke, 2006). The research sought to understand how people who self‐harm perceived their support structures to have changed during the lockdown.

### 2.2 Context

This study was designed and undertaken in collaboration with third‐sector partners who provide support to people who self‐harm, as part of an existing programme of research aiming to support the wellbeing of people who repeatedly self‐harm (NIHR, 2018). Participants were recruited via an advert promoted through social media channels associated with our research programme, the online accounts of third‐sector partners, and self‐harm research networks.

### 2.3 Participants

Participants were included if they were aged 16 or above, living in England, and actively receiving, or attempting to engage with support as a consequence of self‐harm during the first lockdown. We used a semi‐structured topic guide to explore participant experiences of self‐harm and support seeking prior to lockdown, and how their experiences had changed as a consequence of lockdown.

### 2.4 Ethical considerations

We were aware of the ethical implications of carrying out research with people who self‐harm during the pandemic, and the likelihood that people may be managing heightened distress (Townsend et al., 2020). However, feedback from our third‐sector partners suggested that many of those who followed their social media pages were frustrated by a lack of meaningful opportunities to express their concerns with the gap in available support during lockdown. We felt it important to open up such opportunities to those who wished to contribute their experiences, and resolved to do so whilst aware of a need to work with our participants to support their wellbeing during and after the interview.

All participants gave informed consent to take part via a secure online form. As part of a programme of self‐harm focused research, our interviewing researchers were already experienced in talking to people who self‐harm, and received additional training through one of our partner organizations to develop their telephone listening skills. We also employed safeguards with our participants prior to interview, by inviting them to reflect on the personal resources and strategies they would utilize for their safety during or after the interview (or revisiting an existing safety plan) (Cole‐King et al., 2013). Interviewers were able to contact a clinician working within the research team for guidance in the event of sensitive disclosures (i.e. risk of harm to self or others). We additionally provided up‐to‐date contact details for appropriate support agencies following interviews and in our study documentation. Ethical approval to conduct this study was granted by the University of Leeds Research Ethics Committee in June 2020.

### 2.5 Data collection

Twenty‐five people responded to the online advertisement, of whom 14 agreed to take part in an interview. The majority were female (*n* = 11, *n* = 3 male), and White British or Irish (*n* = 12); the remaining two participants were of South Asian heritage. Participants were aged between 21 and 68 years (mean = 35.4 years). The majority of participants opted for telephone contact; one took part via email. Telephone interviews were audio‐recorded and transcribed verbatim. Pseudonyms have been applied to quotes presented in this paper to protect participant anonymity.

### 2.6 Data analysis

We applied thematic analysis (TA) (Braun & Clarke, 2013) on the full set of data. Thematic analysis had advantages with the depth and versatility, allowing us to “go beyond” the surface‐level interpretation of the data to develop a deeper understanding of the lived experiences of our participants.

Our approach to TA followed guidance outlined by Braun and Clarke (Braun & Clarke, 2006, 2013, 2019). Our team (CS, KF, CB) coded a selection of transcripts and compared findings for credibility and validity. Once a coding pattern had been established, the full set were coded and recorded on NVivo (QSR, 2021).

### 2.7 Reflexivity and rigor

Author‐generated codes were developed into overarching themes which were discussed with our stakeholder network. We carried out further development of the thematic headings and relationships between them, before communicating this framework with wider members of the team and experienced stakeholders. These conversations included previous research collaborators with lived experience of self‐harm. Our efforts to communicate our initial thematic structure were necessary to ensure that our conclusions were appropriate and reflective of our participants' realities. Interview participants were invited along with other contacts with lived experience to engage with our findings at an online seminar before finalizing the thematic structure.

## 3 RESULTS

The results will be presented under the two themes generated by the aims of the study, the first “*Adjusting to the changes”* giving context to the impact participants reported as a consequence to the pandemic, and the second, “*Factors affecting help‐seeking during the pandemic”* discussing the factors that affected help‐seeking. In this section, there were three sub‐themes which describe the thoughts, feelings and actions taken by our participants in response to lockdown, “*not wanting to be a burden,” “adjusting to remote communication,” and “worries about contracting the virus.”* Quotations have been utilized within the text, each selected based on an ability to best illustrate the concepts therein (Eldh et al., 2020).

### 3.1 Adjusting to the changes

Participants described how they felt they had coped with the changes to their routine and usual strategies for accessing support. Some found that the change of pace eased feelings of distress in relation to certain factors such as work and relationships, whilst others struggled with the loss of stability, routines and resources, or taking on additional responsibility which negatively impacted their wellbeing. For some, lockdown amplified thoughts of self‐harm.

…the voices started kicking in just before lockdown and then when lockdown happened they got worse because of being on my own and I couldn't go anywhere, I couldn't see anybody so I was like ‘well why not self‐harm, why not listen to the voices because nobody's going to see me’. (Sarah)

Existing domestic conflicts were exacerbated as participants struggled to manage work, study or social relationships all within the home, and many described heightened tensions from feelings of family “all up in my business” (Khalil). Although some participants chose to be closer to family during lockdown, this brought side‐effects such as loss of privacy. Some viewed this as a positive in terms of reducing incidences of self‐harm, as they had fewer opportunities to do so while in the company of others in the home:

I am pleased to say that l have managed not to cut or overdose during lockdown. I think l would have done if l lived alone, but l am aware of the distress it causes to others. Also, my daughter speaks to me every day, and l look forward to spending that time with her. I try to be honest with her, but would find it hard to tell her if l hurt myself. (Denise)

Others struggled with having less time alone, and this could put a strain on relationships which were already tense or difficult prior to the pandemic. Some participants had fewer opportunities to access support from family, friends or others outside the household, especially if they needed a private space to talk candidly about their home situations. Others lived alone and were unable to see other people for prolonged periods, which could lead to feelings of isolation and worsening mental health. Common to all of our participants was the notion that any domestic situation brought challenges in terms of comfort, security and privacy which could potentially affect their strategies for coping with thoughts of self‐harm.

### 3.2 Factors affecting help‐seeking during the pandemic

Participants gave several reasons for feeling less able to seek support for self‐harm during lockdown: wanting to avoid putting additional strain on services; struggling to use alternatives to face‐to‐face contact; or concerns about contracting and spreading COVID‐19 prevented some from reaching out.

#### 3.2.1 Not wanting to be a burden

Under this theme, participants described the feeling of being a burden to others through an awareness of all healthcare services struggling under the pressure of COVID‐19. Such feelings were validated by some individuals failing to meet widening criteria for support, or increasing delays to access.

People who self‐harm may perceive themselves to be a burden to friends and family. The understanding that everyone experienced difficulty during lockdown may have prevented people who self‐harm from asking their friends and family for support out of concern for the people around them. Many of our participants felt they posed a “burden” on services due to their self‐harm and were undeserving of help, and expected that they would experience stigma if they sought support. In lockdown, this was compounded by the messages to stay home and protect others, making them feel even less worthy of support during this time.

No I wouldn't call an ambulance, I know I wouldn't… cos the thing is when you're like that, you already feel like you're a massive burden on people. You know now I can look like rationally and see that I'm not, but when I'm in that kind of state of mind, it's like you don't really have, you just feel like ‘oh I'm such a burden’ like you know ‘I'm going to use an ambulance’. I think, even potentially because of coronavirus people will have not gone, who've got access to A&E, because they would have thought it's needed for other people. (Heather)

Some had experienced situations where their calls for help were not taken seriously by professionals, particularly for those who had managed to cope with alternatives to self‐harm for a long time.

[I've reached] A sort of impasse really (pause). And not really knowing, how to get anybody to take me seriously, because I'm able to articulate how I'm feeling and because I know myself really well, with the kind of things time in the hospital did for me a long time ago, I spent 2 months in hospital and a further 4 months in a full‐time counselling centre afterwards and I probably know myself better than most people and because I'm able to say what I feel is going on and because I've managed my own mental health for 30 years, I feel that I'm being considered as not as poorly. That being successful at managing myself has actually been to my detriment in terms of getting help…and that's hard. (Naomi)

The anticipation of what it would be like to ask for support could be intertwined with negative past experiences, with these heavily influencing expectations of support and capacity to trust health professionals.

Some individuals reported limited or no access to professional support services during lockdown, and others anticipated that their usual sources of support would be inaccessible so took greater pains to manage alone during lockdown. In addition, they worried about adding pressure to services already under strain due to the pandemic.

I think l have found more inner resources during lockdown. It has felt quite scary knowing that help isn't so readily available, and knowing that services are struggling to cope. I feel that l have had no choice but to struggle on. (Denise)

As Denise suggested above, there may be a positive side to attempts to manage without support, such as discovering resilience and building awareness of one's coping style. However, she describes fear at the lack of resources available to her, suggesting that individuals may be at risk if trying to get through lockdown without usual sources of support.

People who self‐harm could harbour feelings of being a burden from repeated experiences of seeking support, and not being the “right fit” for the available provisions. Some reported that this issue had become exacerbated by lockdown due to greater strain on services.

I'm struggling at the moment with trying to get support from local health services and for the first time since December I ended up cutting myself on Friday. So basically I've been told by the mental health specialist at my GP that I don't meet the criteria for a referral to Community Mental Health Team and IAPT [primary care psychological therapy service] aren't responding either. So I'm sort of a bit adrift. (Alice)

Participants experienced issues in accessing consistent, ongoing support from staff who may have been transferred or absent from their previous roles. The breakdown in communication with professional services may have been exacerbated by restrictions introduced to limit virus transmission.

I guess the big thing in terms of the support that I had is my co‐coordinator is asthmatic so she had to stay at home for 12 weeks, which, I mean she was working from home, so we spoke on the phone, but erm that was quite hard, er, definitely wasn't the same. (Grace)

The widespread shift from face‐to‐face to remote contact led some participants to feel disconnected and confused by the process.

I would like to have got an email to say, this is how you contact us if you need to, you know like please do contact us if you need to, that hasn't really happened. (Anthony)

Some lost the ability to keep track of availability of existing support, or progress on waiting lists, and others awaiting support felt they might be lost in the system through becoming further removed from the process. The experiences summarized above illustrate the ways in which individuals may feel further validated in thoughts of being a burden on healthcare providers and personal support networks during lockdown. Despite such feelings, some participants persevered with remote modes of contact to continue communication with social and professional support networks, and their experiences are explored under the next sub‐theme.

#### 3.2.2 Adjusting to remote communication

Remote communications could warrant a change in the existing boundaries or rules of engagement that were familiar to participants during face‐to‐face contact with professionals. Without clear expectations and parameters, participants could feel uncertain and described the nature of their support from professionals changing, feeling like they were no longer able to discuss their needs in the same depth as before.

Our participants told us about the nature of support that they required during lockdown. Telephone and online listening services provided through third‐sector organizations, although readily available throughout lockdown, were not seen as an adequate replacement for formal support services such as those delivered through the NHS. Participants perceived a lack of understanding of self‐harm, and limited ability to offer practical support during a crisis.

So if it's anything to do with self‐harm … I wouldn't ring Samaritans, I would ring my CPN or the GP surgery … I think because the type of support they could offer. I don't think these listening services are well geared and prepared around supporting people with self‐harm behaviour, because they have very limited knowledge or, very limited understanding around what some of the challenges are or what some of the issues they are presented with … Just with the GP there would be more support in terms of … what type of service we could refer them to or what could be helpful in the case where they can assist the individual. (Hakim)

Of the participants offered formal support online for self‐harm, some experienced issues to access, recalling occasions where technology faults had created barriers to working with professionals or communicating with social networks. When people who self‐harm wished to engage in online support or therapy, they may have felt uncomfortable opening up and engaging in the intervention. Building a therapeutic alliance online, given that both parties may not have met in person, or be unfamiliar in the use of video calls, required a period of adjustment. Participants reported that video calls lacked non‐verbal feedback and opportunities to build rapport with their therapists, and the use of unknown platforms brought concerns related to confidentiality. Some also struggled to communicate during group calls with multiple people vying for the opportunity to speak, and noted that social conventions were different online, with many preferring cameras or microphones to remain off and create an additional barrier to engagement.

We go to a waiting room, an online waiting room, and you just wait until your worker picks up the call and then it's just a video. [The experience is] weird. Because with my DBT nurse, I just started it and then, the coronavirus kicked in so my nurse was deployed elsewhere so I found it totally new to me, so that was the first time I met her was on the video link. So it was a bit strange, it was a bit unknown and I don't know this person. (Sarah)

Participants also reflected on the impact of changing the environment in which support took place. For some, their homes (or a private area within the home) represented a valuable place of safety. In communicating online with others within these spaces, this felt to some like an intrusion or invasion of privacy, or could alter the safe atmosphere within that space. Others missed the opportunity to decompress from support or therapy, because of no longer travelling between meetings.

It feels kind of invasive in a way, it's in your home, I'm quite territorial about my flat because it took me a long time to have anywhere that was private, just for particular practicalities and the nature of my relationship with my family, I feel like I've had to fight quite hard for space for myself. And the idea of having therapy where you're talking about really difficult stuff and then you're left with that in your home … even though travelling when you're upset is really hard, it does give you a bit of time … to put some of what you've talked about behind you in a way. (Grace)

Some participants reported that services had been cautious about offering video or telephone contact to them in exchange for face‐to‐face support during the pandemic, suggesting they were uncomfortable managing risk from a distance. This meant that some were left without support altogether.

I have researched and stuff into groups and online therapy but I'm not suitable for online therapy sadly, I've looked into self‐harm stuff, and support … I've had seven suicide attempts and actively suicidal, they see it more as a ‘you need to contact somebody you can see in person’. Instead of actually online therapy (Emma)

Despite obvious frustrations, people who self‐harm acknowledged the issues faced by professional support services in meeting the needs of individuals during the pandemic. Their expectations related predominantly to not being forgotten, and continuing to receive reasonable amounts of support where possible. This helped them to feel like they still mattered in spite of shifted priorities, especially with strained NHS services:

In terms of … talking to a GP, I think I've probably had more time in lockdown than I would normally. The phone call conversations I think have been more at length. (Alice)

Others appreciated the opportunity to utilize support offering text or email during the pandemic, reporting this to be easier in some situations for expressing thoughts and feelings compared with the intensity of face‐to‐face support from other people.

I don't know really I think that, I find it stressful kind of getting support at all really and it's a bit easier if you're doing it via text or messaging or something (Anthony)

#### 3.2.3 Worries about the virus

Worries about being exposed to COVID‐19, and causing harm to others by transmitting the virus, manifested in different ways. Despite making efforts to cope without accessing support, some experienced a deterioration in their mental health, continuing to self‐harm and potentially seeking crisis support at a later stage.

I was really struggling, I was really trying to avoid either ending up in a medical hospital or having an admission to a crisis house or a psychiatric ward because, it just made me so anxious the infection stuff, and I think that just meant that all just contributed to my eating getting really difficult again in a way that it hasn't been for several years… I mean, it didn't really work because I did end up in hospital and in a crisis house but it did delay it. It's just that now I've had a crisis and I've got an eating disorder again so, not ideal. (Grace)

Others were able to use the situation to stop self‐harm, out of concern for the impact on other people. The thought of being responsible for transmitting the virus to others could exacerbate existing feelings of shame, or of being unworthy of help for self‐harm.

l know the hospitals are particularly busy, and l would not want to put others at risk in my family if l picked something up from the hospital. My husband is shielding, so l am being particularly careful. Once when l was in casualty, there were patients waiting in corridors, and l felt so bad about it that l determined not to hurt myself so badly that l would need to take up a hospital place. (Denise)

Despite concerns about virus transmission, some were keen to accept face‐to‐face support where available, for example, if they perceived this as essential for their wellbeing. They described strategies for weighing up the acceptability of risks, to ensure they received the support they needed:

I can see my mental health team face to face now, erm, although travelling there is quite difficult, because it means getting the bus and I'm quite anxious about infection on public transport, so I'm not going every week to see them face to face but I am some of the time and that does help. (Grace)

Participants acknowledged the difficulties professionals were facing with trying to keep their services running, and were thankful for the chance to stay connected, helping to remind them that they had a lifeline in times of crisis.

In spite of an identified need for support and the challenges associated with access in lockdown, participants internalized public guidance to reduce the burden on the NHS and crisis support services (“not wanting to be a burden”), potentially increasing their personal risk of escalating mental ill‐health in the process. They grappled with new challenges posed by a shift to remote communication by many existing services, working to understand different boundaries and rules of engagement (“adjusting to remote communication”). Finally, our participants described the varied ways in which the pandemic affected their help‐seeking behaviours related to self‐harm (“worries about contracting the virus”).

## 4 DISCUSSION

Our findings from interviews with people who self‐harm during the initial UK lockdown period demonstrate the diversity of experiences and interplay of positive and negative aspects of life during the pandemic. There is an overall lack of evidence to indicate changes in prevalence of self‐harm in the population (John et al., 2020) in spite of a decline in self‐harm related presentations in hospital in England (Hawton, Casey, et al., 2021). Our findings should indicate that a reduction in contact with support services does not necessarily mean that levels of distress and self‐harm reduced during the pandemic, a phenomenon which researchers have indicated elsewhere (NIHR, 2021a, 2021b). Lockdown presented difficulties for people who self‐harm, exacerbating feelings of loneliness and isolation for some, while others faced increased tensions with family in home environments. Such feelings have also been cited as precipitating factors for self‐harm by those attending emergency departments in hospital for support (Hawton, Lascelles, et al., 2021). These concerns aligned with the most impactful difficulties reported by people with mental health conditions in the early stages of the pandemic (Rains et al., 2021), and were associated with loneliness, feeling overwhelmed, loss of preferred coping mechanisms (e.g. social or leisure activities), frustration and anger. Existing support structures affected how individuals felt able to access support. Participants acknowledged the lack of alternative strategies to coping with lockdown beyond self‐harm, leading to a reluctance among some individuals to disclose their feelings to others in case someone tried to stop them. Previous studies have emphasized the role of interpersonal relationships on self‐harm, with concerns about other people serving as a mechanism for cessation (Brennan et al., 2022). Although existing literature advocates for modifying a person's social environment to counteract such issues, it is important to acknowledge that such changes may be harder to enact given the instability of many individual's circumstances during the pandemic. This emphasizes the importance of an understanding of self‐harm and the varied functions this could serve during a time of limited opportunities for social contact.

In spite of these challenges, our participants reported an increase in the volume of interactions with existing social networks during lockdown. This reflects other research findings indicating that people experiencing thoughts of self‐harm were in touch with friends and family more often than before the pandemic (Iob et al., 2020). This could be a consequence of the loss of formal support, and an increased focus on connecting with others through remote platforms, such as voice and video calling. However, as some of our participants demonstrated, they may have stayed reluctant to communicate their innermost feelings. Other researchers have considered that the universal struggle of lockdown may have intensified fears of being a burden to others (Rains et al., 2021). This necessitates initiatives which encourage greater quality of communication between families and friends, and educate the public to understand self‐harm and how to support those who are affected.

Participants internalized messages to protect the NHS; which combined with expected shame or stigma from healthcare services and concerns about exposure to the virus, contributed to efforts to stay out of hospital. This helped some to reduce self‐harm, but led others to delay treatment and run into crisis. For some, the avoidance of services may have been a protective mechanism against the potential disappointment from reaching out and finding that nothing was available to help them. Some imposed their own assumptions of the availability of services or the need to protect others from the virus. Those who did use services found that the support offered had changed, and faced new barriers to engaging with professionals. Some may have missed out on support because they were too unwell for some services, and not enough for others (Saunders & Smith, 2016), highlighting gaps in availability of services for people who fall in‐between low intensity and crisis services. This may have forced some to cope alone or wait until their conditions worsen. There is also shortage of effective interventions to support individuals in reducing or stopping self‐harm, without considering the additional barriers to integrating these approaches with remote forms of communication (Hawton et al., 2016).

Our findings suggest that practices to curb transmission of COVID‐19 may have reduced engagement with therapeutic support, which aligns with existing research carried out early in the pandemic (Rains et al., 2021). Communication barriers such as poor internet or lack of privacy could affect whether support was beneficial. Some expressed reservations around trust and safety, inhibiting their ability to express themselves freely. Whilst online resources were regarded as helpful by our participants, these are not created equally and even websites associated with leading support organizations may be ineffective in engaging those in crisis. Evaluations of self‐harm support websites have shown that many sites are unable to provide a direct response to a user in crisis, and may signpost to offline services (Biddle et al., 2020). People accessing such resources require responsive communication, lived experience accounts and useful self‐help strategies.

Self‐harm can serve a multitude of different purposes for individuals, both positive and negative (Edmondson et al., 2016). Our findings suggested a protective function of self‐harm during lockdown, in the absence of some existing and vital support provisions. Although lockdown posed many challenges to people who self‐harm, they also reported positive outcomes and found ways of coping. Some found time to focus on finding new strategies to improve their mental health. They controlled their news intake, and took advantage of opportunities to connect with friends and family on a deeper level. Similar positive outcomes have been reported elsewhere (Gillard et al., 2021; Rains et al., 2021). Rains and colleagues also found positive factors such as a shared trauma, the ability to use existing resilience and positive ways of coping, and a more widespread use of remote technologies could help some to connect better with the outside world. They accepted the difficulty of the situation for formal support services, and valued the effort made by professionals to maintain communication during lockdown.

### 4.1 Implications

In light of the potential risks to individuals who avoided seeking support for self‐harm during the pandemic, it is important to consider ways in which people who self‐harm may be supported to understand what support is available to them during periods of restrictions and lockdown. Guidance for health services around communicating important health messages and guidance with the public is also essential, whilst acknowledging that some individuals are susceptible to feelings of shame in relation to help‐seeking and this may prevent them from accessing timely support. This has implications for initiatives such as “111 first” whereby individuals are encouraged to pre‐book visits to hospital emergency departments (Healthwatch, 2020).

Professionals may value the opportunities created through online platforms, for efficiency, flexibility, and enabling services to continue during lockdown. The experiences reported in this study, suggest services providing support for people who self‐harm should ensure staff have the resources, skills and confidence to use online services to improve accessibility of support. Additional time and effort should be devoted to establishing a therapeutic alliance and ensuring individuals have access to a suitable, private space. Remote support may be inappropriate for some individuals, and it is imperative that support services avoid widening inequalities for accessing support (Johnson et al., 2020; Townsend, 2020).

Academics have cautioned against burdening people with mental health conditions with participation in research during this challenging time (Townsend et al., 2020). Through conversations with our third‐sector stakeholders, we were encouraged by the determination of people within their networks to share experiences with service providers, in the aim of improving future practice and opening up avenues for research. We believe this study gave a platform for meaningful contribution to the knowledge base, in support of those facing frustrations and adversity.

### 4.2 Limitations

Our recruitment took place online during lockdown; therefore, we could not invite individuals without internet access or social media accounts to participate. We were limited in our ability to advertise and engage with our intended audience. However, we utilized opportunities to promote the study through support organizations with wide‐reaching networks of people with lived experience, and offered email participation to include individuals who were uncomfortable with telephone interviews. Email interviews carried a higher chance of drop‐out over the course of the exchange compared with a single telephone call, which could result in some missed data. Feedback from those taking part suggested they had space to consider their responses with greater care and depth than during a one‐off telephone call with a researcher. Participants also described a rare opportunity to reflect on important personal issues both during the course of the interview, and in the future, as they were able to retain a copy of their replies. Few who took part were from minority ethnic backgrounds, which may have been a consequence of the limited time and engagement opportunities we could undertake with partner organizations.

## 5 RELEVANCE STATEMENT

The most common point of contact with acute healthcare services for people who self‐harm is the emergency department. Mental health nurses working within mental health liaison services work most closely with these patients, triaging, assessing and referring patients into further support services. Understanding how patients perceive these services will help mental health nurses to engage with and support this population. The experiences captured by this study will support mental health liaison nurses to understand the impact of national lockdown on this vulnerable group.

## ETHICAL APPROVAL

This study was granted approval by the University of Leeds School of Medicine Research Ethics Committee (SoMREC) in June 2020. Reference: MREC 19‐083.

## Data Availability

Data available on request due to privacy/ethical restrictions.
